# Cardiac Fas-Dependent and Mitochondria-Dependent Apoptosis after Chronic Cocaine Abuse

**DOI:** 10.3390/ijms15045988

**Published:** 2014-04-09

**Authors:** Cher-Ming Liou, Shiow-Chwen Tsai, Chia-Hua Kuo, Hua Ting, Shin-Da Lee

**Affiliations:** 1 Department of Anesthesiology, Chung Shan Medical University Hospital, Taichung 402, Taiwan; 2 Institute of Medicine, Chung Shan Medical University, Taichung 402, Taiwan; E-Mails: liouk04.peter@msa.hinet.net (C.-M.L.); huating@csmu.edu.tw (H.T.); 3 Department of Sports Sciences, University of Taipei, Taipei 111, Taiwan; E-Mail: sctsai6@gmail.com; 4 Laboratory of Exercise Biochemistry, University of Taipei, Taipei 111, Taiwan; E-Mail: kuochiahua@gmail.com; 5 Department of Physical Medicine and Rehabilitation, Chung Shan Medical University Hospital, Taichung 402, Taiwan; 6 Department of Physical Therapy, Graduate Institute of Rehabilitation Science, China Medical University, Taichung 402, Taiwan; 7 Department of Healthcare Administration, Asia University, Taichung 413, Taiwan; 8 School of Rehabilitation Medicine, Shanghai University of TCM, Shanghai 201203, China

**Keywords:** heart, apoptotic, cocaine, caspase, cardiotoxicity

## Abstract

To evaluate whether chronic cocaine abuse will increase cardiac Fas-dependent and mitochondria-dependent apoptotic pathways, thirty-two male Wistar rats at 3–4 months of age were randomly divided into a vehicle-treated group (phosphate-buffered saline, PBS, 0.5 mL, SQ per day) and a cocaine-treated group (Cocaine, 10 mg/kg, SQ per day). After 3 months of treatment, the excised left ventricles were measured by H&E staining, Western blotting, DAPI staining and TUNEL assays. More cardiac TUNEL-positive apoptotic cells were observed in the Cocaine group than the PBS group. Protein levels of TNF-alpha, Fas ligand, Fas death receptor, FADD, activated caspase-8, and activated caspase-3 (Fas-dependent apoptosis) extracted from excised hearts in the Cocaine group were significantly increased, compared to the PBS group. Protein levels of cardiac Bax, cytosolic cytochrome *c*, t-Bid-to-Bid, Bak-to-Bcl-xL, Bax-to-Bcl-2 ratio, activated caspase-9, and activated caspase-3 (mitochondria-dependent apoptosis) were significantly increased in the Cocaine group, compared to the PBS group. Chronic cocaine exposure appeared to activate the cardiac Fas-dependent and mitochondria-dependent apoptosis, which may indicate a possible mechanism for the development of cardiac abnormalities in humans with chronic cocaine abuse.

## 1. Introduction

Cocaine is the most commonly used illicit drug in the world. Cardiovascular complications related to cocaine abuse include myocardial ischemia, infarction, inflammation, rhythm disturbances, aortic dissection and sudden cardiac death [1–4]. Long-term cocaine abuse has been reported to cause left ventricular hypertrophy, dilated cardiomyopathy and systolic dysfunction [4–8].

Apoptosis, a physiological program of cellular death, may contribute to many cardiac disorders [9,10]. Apoptosis has been reported to contribute to the loss of cardiomyocytes in cardiomyopathy, and is recognized as a predictor of adverse outcomes in patients with cardiac diseases or heart failure [11]. Consequently, the interruption of apoptotic pathways could allow development of novel strategies to reverse or attenuate heart failure [12,13]. The Fas-dependent apoptotic pathway is believed to be one of the major pathways directly triggering cardiac apoptosis [9,14,15]. This pathway is often initiated by the Fas ligand or the tumor necrosis factor-alpha (TNF-α), which initiate the extrinsic pathway [14]. Fas ligand (Fas L) binding followed by Fas-receptor (Fas) oligomerization leads to formation of a death-inducing signal complex starting with recruitment of the Fas-associated death domain (FADD) of the adaptor protein [14]. FADD is known to function as a common signaling conduit in both Fas and TNF-α mediated apoptosis [16]. FADD recruits and aggregates the pro- form of caspase-8, leading to the activation of caspase-8 [15]. The activated caspase-8 could cleave pro-caspase-3, which then undergoes autocatalysis to form active caspase-3, a principle effector caspase of apoptosis [17].

The mitochondria-dependent apoptotic pathway starts from within the cell resulting in the release of a number of pro-apoptotic factors from the intermembrane space of mitochondria [14,15]. The mitochondria is the main site of action for members of the apoptosis-regulating protein family, exemplified by the Bcl-2 (B-cell lymphoma 2) family, such as Bak (Bcl-2 homologous antagonist/killer), Bcl-2, Bax (Bcl-2-associated X protein) and Bad (Bcl-2-associated death promoter) [14]. Commitment to apoptosis is typically governed by opposing factions of the Bcl-2 family, including pro-apoptotic *vs.* anti-apoptotic family members [18]. Pro-apoptotic and anti-apoptotic Bcl-2 family members can homodimerize or heterodimerize to each other, and appear to interact with and neutralize each other, so that the relative balance of these effectors strongly influences cytochrome *c* release and cell fate [19]. Bcl-2, p-Bad (phosphate-Bcl-2-associated death promoter) and Bcl-xL (B-cell lymphoma-extra large) prevent cytochrome *c* release whereas Bad, Bak and Bax enhance cytochrome *c* release from mitochondria [14]. When cytochrome *c* is released from mitochondria into the cytosol, it is responsible for activating caspase-9, which further activates caspase-3 and executes the apoptotic program [20]. Additionally, caspase-8 can cleave Bid (Bcl-2 homology domain 3 (BH3) interacting domain death agonist) into truncate Bid (t-Bid), then cause the release of mitochondrial cytochrome *c*, leading to the activation of caspase-9, which can then activate caspase-3 [14,21,22]. Bid is one of the key components of main intracellular molecule signaling from Fas to the mitochondrial apoptotic pathway [14,21]. However, the mechanism of cardiac apoptosis in cocaine-treated animal models remains unclear.

Previous studies have suggested that cocaine-induced apoptosis in cardiomyocytes is mediated only by the mitochondria-dependent pathway in the cell line [1,3]. The current study was undertaken to understand whether cardiac Fas-dependent and mitochondria-dependent apoptosis could be determined from the excised cardiac tissues of the PBS and Cocaine treated rats. We hypothesized that chronic cocaine exposure in rats may predispose them to greater levels of activated cardiac Fas-dependent and mitochondria-dependent apoptosis.

## 2. Results and Discussion

### 2.1. Body Weight and Cardiac Characteristics

Body weight (BW), whole heart weight (WHW), and the ratio of whole heart weight to body weight (WHW/BW) were not significantly different between the PBS and Cocaine groups (Table 1).

### 2.2. Cardiac Histopathological and Apoptotic Cells Changes

**Figure 1.**
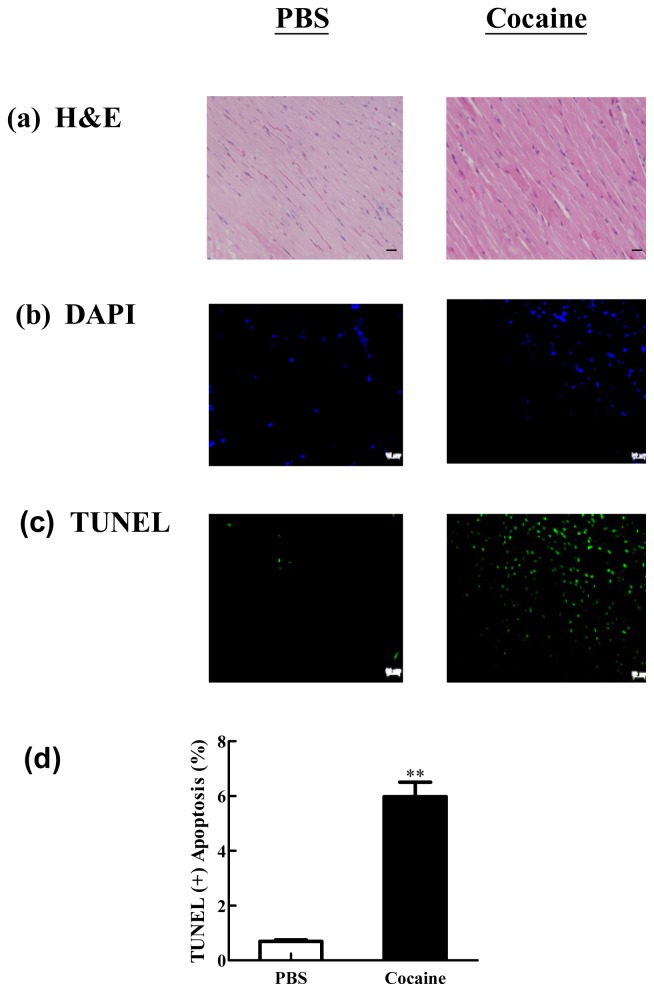
Representive analysis of cardiac tissue sections with (**a**) Hematoxylin and eosin staining (H&E), The images were magnified 100 times; Bars scales = 50 μm; (**b**) 4′,6-diamidino-2-phenylindole (DAPI) staining; (**c**) Terminal deoxynucleotidyl transferase UTP Nick End Labeling (TUNEL) assay in Wistar rats injected with PBS (PBS) and Wistar rats injected with cocaine (Cocaine). The images were magnified 100×; (**d**) Bars present the percentage of TUNEL positive cells relative to total cells (6 rats × 30 scope field counts in each group). ** *p* < 0.01, significant differences between PBS and Cocaine.

To investigate myocardial architecture after chronic cocaine exposure, a histopathological analysis of ventricular tissue of both PBS and Cocaine groups was performed with hematoxylin and eosin staining. After viewing 100× magnified images, we found that the ventricular myocardium in the PBS group indicated normal architecture with normal interstitial spacing, while, in contrast, abnormal myocardial architecture and interstitial spacing were observed in the Cocaine group. In order to view the cardiac apoptotic activity after chronic cocaine exposure, the TUNEL-positive cardiac cells were examined in the PBS and Cocaine groups by DAPI staining and TUNEL assay. By viewing images magnified 100×, we observed that the left ventricle stained with the TUNEL assay showed increased TUNEL-positive cardiac cells in the Cocaine group, compared with the PBS group (0.69 ± 0.08 *vs.* 5.97 ± 0.74) (Figure 1).

### 2.3. Upstream Components of Cardiac Fas Receptor Dependent Apoptotic Pathways

**Figure 2.**
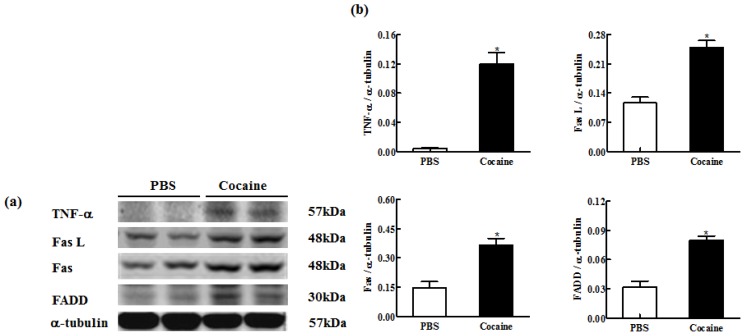
(**a**) The representative protein products of tumor necrosis factor-alpha (TNF-α), Fas ligand (Fas L), Fas receptor (Fas) and Fas-associated death domain (FADD) extracted from the left ventricles of excised hearts in 2 Wistar rats with PBS exposure (PBS), 2 Wistar rats with cocaine exposure (Cocaine) as measured by Western blotting analysis. α-tubulin was used as an internal control; (**b**) Bars represent the relative changes of protein quantification on the basis of α-tubulin in TNF-α, Fas L, Fas and FADD and indicate mean values ± SD (*n* = 6 in each group). * *p* < 0.05, significant differences between PBS and Cocaine.

To investigate the upstream components of cardiac Fas-dependent apoptotic signaling pathways in the Cocaine group, the protein levels of TNF-α, Fas ligand, Fas receptor and FADD in the excised hearts of both the PBS and Cocaine groups were examined by Western blotting. The protein levels of TNF-α (0.003 ± 0.003 *vs.* 0.110 ± 0.036), Fas ligand (0.116 ± 0.019 *vs.* 0.239 ± 0.038), Fas receptor (0.147 ± 0.044 *vs.* 0.365 ± 0.050) and FADD (0.032 ± 0.008 *vs.* 0.075 ± 0.013) were significantly higher in the Cocaine group compared with the PBS group (Figure 2).

### 2.4. Main Intracellular Molecule Signaling Mediator from Fas to Mitochondrial Pathway

**Figure 3.**
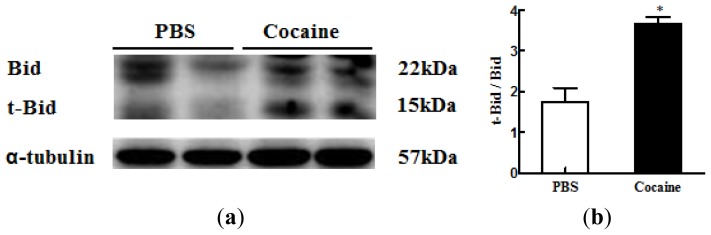
(**a**) The representative protein products of BH3 interacting domain death agonist (Bid), truncated Bid (t-Bid) extracted from the left ventricles of excised hearts in 2 Wistar rats with PBS exposure (PBS), 2 Wistar rats with cocaine exposure (Cocaine) as measured by Western blotting analysis. α-tubulin was used as an internal control; (**b**) Bar represents the ratio of t-Bid to Bid and indicates mean values ± SD (*n* = 6 in each group). * *p* < 0.05, significant difference between PBS and Cocaine.

To investigate the cardiac Bid cleavage, a mediator that connects the Fas-dependent apoptosis pathway to the mitochondria-dependent apoptosis pathway in the Cocaine group, we examined the protein levels of Bid and t-Bid in both the PBS and Cocaine groups by Western blotting. The ratio of t-Bid to Bid was significantly increased (1.938 ± 0.773 *vs.* 3.652 ± 0.249) in the Cocaine group, compared with the PBS group (Figure 3).

### 2.5. Upstream Components of Cardiac Mitochondria-Dependent Apoptotic Pathways

**Figure 4.**
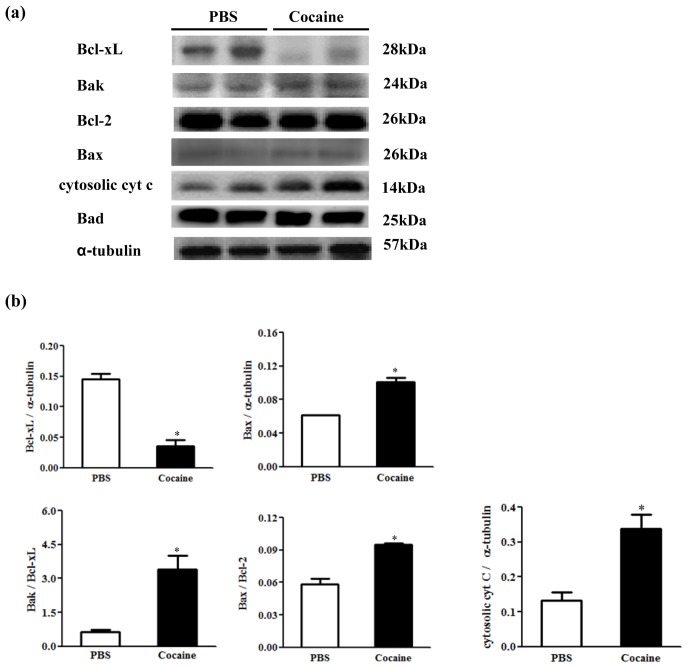
(**a**) The representative protein products of B-cell lymphoma-extra large (Bcl-xL), Bcl-2 homologous antagonist/killer (Bak), B-cell lymphoma 2 (Bcl-2), Bcl-2–associated X protein (Bax), cytosolic cytochrome *c* (cytosolic cyt *c*) and Bcl-2–associated death promoter (Bad) extracted from the left ventricles of excised hearts in 2 Wistar rats with PBS exposure (PBS), 2 Wistar rats with cocaine exposure (Cocaine) as measured by Western blotting analysis. α-tubulin was used as an internal control; (**b**) Bars represent the relative changes of protein quantification on the basis of α-tubulin in Bcl-xL, Bax and cytosolic cytochrome *c* or the ratios of Bak to Bcl-xL, Bax to Bcl-2 and indicate mean values ± SD (*n* = 6 in each group). * *p* < 0.05, significant differences between PBS and Cocaine.

To further understand the cardiac Bcl-2 family in the mitochondria-dependent apoptotic pathway in the Cocaine group, we examined the protein levels of the Bcl-2 family (Bcl-xL, Bak, Bcl-2, Bax, Bad) and cytosolic cytochrome *c* (cytochrome *c* released from mitochondria) in the excised hearts of both the PBS and Cocaine groups by Western blotting. Mitochondrial related pro-apoptotic proteins of Bax (0.062 ± 0.000 *vs.* 0.096 ± 0.014), cytochrome *c* (0.202 ± 0.054 *vs.* 0.437 ± 0.201), the ratio of Bak/Bcl-xL (0.644 ± 0.107 *vs.* 3.396 ± 0.866), Bax/Bcl-2 (0.058 ± 0.007 *vs.* 0.095 ± 0.002), were significantly increased in the Cocaine group; while anti-apoptotic protein Bcl-xL (0.135 ± 0.028 *vs.* 0.035 ± 0.015) was significantly decreased, compared with the PBS group (Figure 4). The quantification for bak/α-tubulin (0.085 ± 0.004 *vs.* 0.083 ± 0.022) and for Bcl2/α-tubulin is (0.998 ± 0.229 *vs.* 1.011 ± 0.120) were similar between PBS and cocain group.

### 2.6. Downstream Components of Cardiac Fas-Dependent and Mitochondria-Dependent Apoptotic Pathways

**Figure 5.**
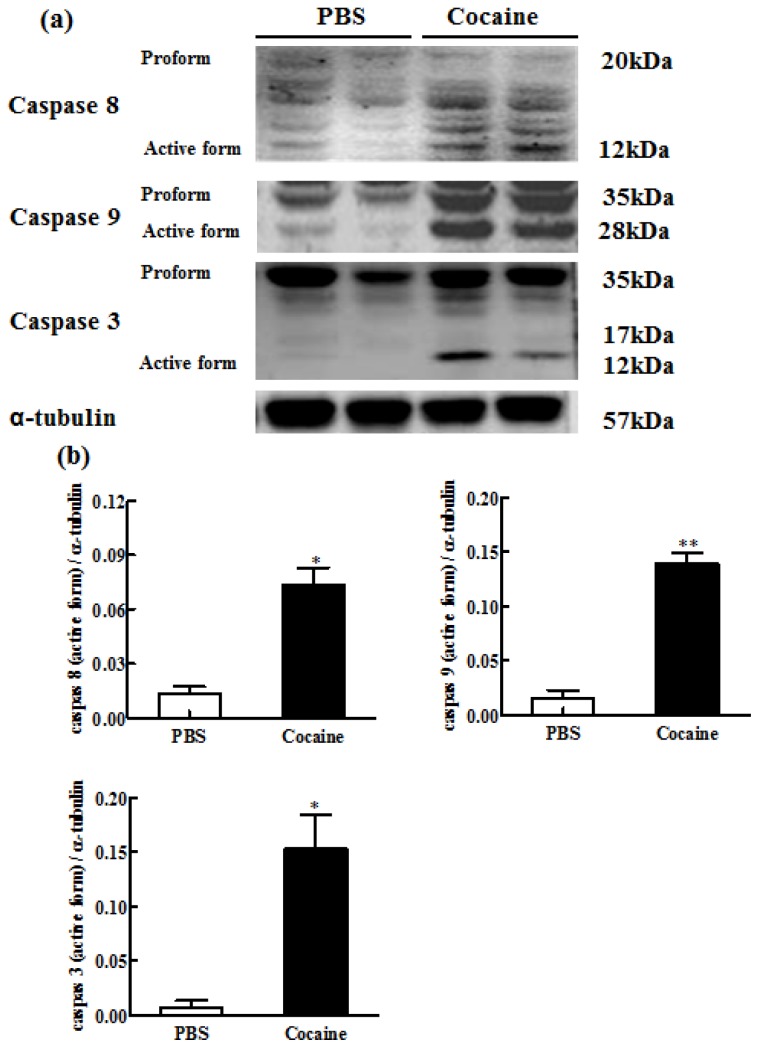
(**a**) The representative protein products of caspases-8, -9 and -3 extracted from the left ventricles of excised hearts in 2 Wistar rats with PBS exposure (PBS), 2 Wistar rats with cocaine exposure (Cocaine) as measured by Western blotting analysis. α-tubulin was used as an internal control; (**b**) Bars represent the relative changes of protein quantification on the basis of α-tubulin in activated caspase-8, activated caspase-9 and activated caspase-3 and indicate mean values ± SD (*n* = 6 in each group). * *p* < 0.05, ** *p* < 0.01, significant differences between PBS and Cocaine.

To identify the downstream components of cardiac Fas (caspases-8 and -3) and mitochondria (caspases-9 and -3) dependent apoptotic pathways, the protein levels of activated caspases-8, -9 and -3 were measured in the excised hearts of both the PBS and Cocaine groups by Western blotting. Activated caspase-8 (0.011 ± 0.009 *vs.* 0.063 ± 0.028), caspase-9 (0.016 ± 0.010 *vs.* 0.139 ± 0.014), and caspase-3 (0.004 ± 0.006 *vs.* 0.118 ± 0.094) protein products were significantly increased in the Cocaine group, compared with the PBS group (Figure 5).

**Figure 6.**
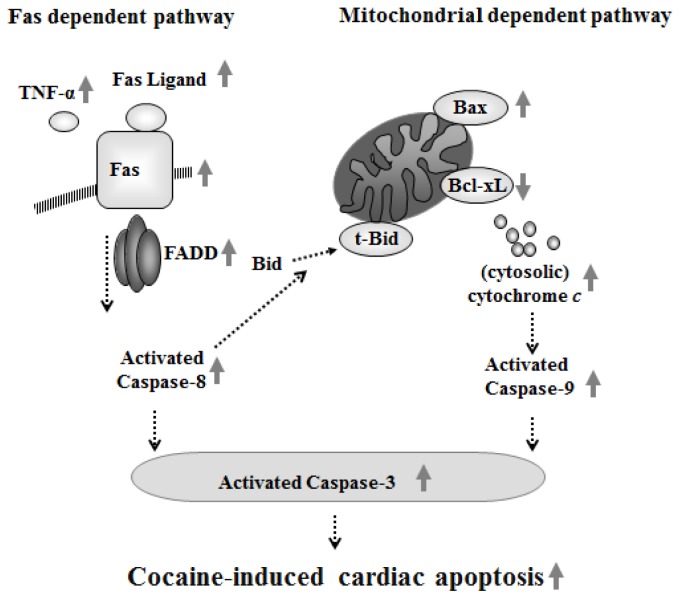
Our proposed major hypothesis: cardiac Fas-dependent and mitochondrial-dependent apoptotic pathways might be more activated after chronic cocaine administration. Cardiac Fas-dependent apoptotic pathway increased due to increases in TNF-α, Fas ligand, Fas, FADD, activated caspase-8, and activated caspase-3. Cardiac mitochondria-dependent apoptotic pathway increased due to decreases in Bcl-xL and increases in t-Bid/Bid, Bax, Bak/Bcl-xL, Bax/Bcl-2, cytochrome *c* release, activated caspase-9, and activated caspase-3. Up arrows and down arrows on the right side represent increases and decreases, respectively.

Our main findings can be summarized as follows: (1) the body weight, whole heart weight, and the ratio of whole heart weight to body weight were not significantly different between the PBS and Cocaine groups; (2) Abnormal myocardial architecture, increased interstitial space, and a greater number of TUNEL-positive apoptotic cells were observed in the Cocaine exposed rats relative to PBS group; (3) The cardiac Fas-dependent apoptotic pathway was significantly more activated in the Cocaine group relative to the PBS group, the evidence for which is based on increases in TNF-alpha, Fas ligand, Fas death receptors, FADD, activated caspase-8, and activated caspase-3 (Figure 6); (4) The cardiac mitochondria-dependent apoptotic pathway was significantly more activated in the Cocaine group relative to PBS group, the evidence for which is based on increases in t-Bid/Bid, Bax, Bak/Bcl-xL, Bax/Bcl-2, cytosolic cytochrome *c*, activated caspase-9, and activated caspase-3 (Figure 6).

Exposure to cocaine can be acquired orally, subcutaneously, intraperitoneally, intravenously, intranasally, sublingually, intravaginally, or rectally [23–25]. The treatment regime of cocaine at 10–60 mg/kg/day has been used in many studies, and in most studies, the time-points for observation after cocaine injections were from 2 to 15 days [24–26]. In the current experimental design, cocaine was injected subcutaneously because it seemed to be less stressful than intraperitoneally [27]. In the current study, the time period of three months for cocaine treatment at a dosage of 10 mg/kg was chosen to observe the long term effects of cocaine abuse on cardiac health, rather than the short term effects.

Cardiovascular complications related to cocaine abuse include non-ischaemic myocardial damage, myocardial ischemia, infarction, myocarditis, rhythm disturbances, aortic dissection and sudden cardiac death [1–4]. Chronic cocaine abuse can cause left ventricular hypertrophy, dilated cardiomyopathy, profound myocardial depression and systolic dysfunction [4–8,28]. The cardiomyopathic changes found in the current study may support previous pathophysiologic mechanisms in people with chronic cocaine abuse.

Previous studies have suggested that cocaine-induced apoptosis in cardiomyocytes is mediated only by the mitochondrial pathway in cell lines [1,3]. Cocaine induced a translocation of cytochrome *c* from the mitochondria to the cytosol [29]. Correspondingly, cocaine induced activation of caspase-9 preceded that of caspase-3, but caspase-8 was not activated [29–31]. The cardiac activity of the Fas receptor-dependent apoptotic pathway after chronic cocaine exposure has never been clarified before the current study. In the current study, all key components of Fas receptor-dependent apoptotic pathway from upstream cascade (increases in TNF-alpha, Fas ligand, Fas receptor, FADD) to downstream cascade (increases in activated caspase-8, and activated caspase-3) consistently demonstrate the pro-apoptotic effects of cocaine exposure, as well as an increase in cardiac TUNEL-positive apoptotic cells. Therefore, our findings suggest that chronic cocaine exposure might activate the cardiac Fas receptor-dependent apoptotic pathway and cause cardiac apoptosis. This is the first paper to show the Fas-dependent apoptotic pathway in the animal model of cocaine abuse. Because caspase-8 was not activated in the cardiac cell line [24,29–31], the Fas-dependent pathway might be activated via systemic effects instead of direct effects on cells.

Pro-apoptotic and anti-apoptotic members of the Bcl-2 family appear to interact with and neutralize each other, so that the relative balance of these effectors strongly influences cell fate [19]. In other words, the up regulation of the pro-apoptotic Bcl-2 family; Bad, Bak, Bax, cytochrome *c* and the increased apoptotic index, t-Bid/Bid, Bak/Bcl-xL, Bax/Bcl-2, are possible indicators of a shift in the balance between anti-apoptotic effects and pro-apoptotic effects towards the latter. That the mitochondria-dependent apoptotic pathway was significantly enhanced in Cocaine exposed rats may be determined from evidence such as; increases in Bax, t-Bid/Bid, Bak/Bcl-xL, Bax/Bcl-2 ratio, cytosolic cytochrome *c* (upstream cascade), activated caspase-9, and activated caspase-3 (downstream cascade). The current findings of chronic cocaine treatment in an animal model and our previous study with chronic methamphetamine treatment both show similar pro-apoptotic effects with different activation of Bcl2 family, such as Bax and Bad [32]. Therefore, our findings strongly suggest that chronic cocaine exposure will activate the cardiac mitochondria-dependent apoptotic pathway, and cause cardiac apoptosis.

Since cardiac tissues are difficult to extract from human hearts, the current cocaine-treated animal model should provide an important mechanism for explaining cardiac diseases in people with chronic cocaine abuse [2,4–8,28]. If cardiac apoptosis occurs in a chronic cocaine user, long-lasting and extensive cardiac apoptosis may progressively develop into heart failure and sudden cardiac death. Our current findings indicating “a greater activated cardiac Fas- and mitochondria- dependent apoptotic pathway after chronic cocaine exposure” might provide one possible mechanism to explain the development of heart failure in chronic cocaine users.

## 3. Experimental Section

### 3.1. Animals

Thirty two 3–4 month old male Wistar rats were purchased from the National Laboratory Animal Center, Taiwan. Ambient temperature was maintained at 25 °C and the animals were kept on an artificial 12-h light-dark cycle. The light period began at 7:00 a.m. Rats were provided with standard laboratory chow (Lab Diet 5001; PMI Nutrition International Inc., Brentwood, MO, USA) and water ad libitum. Each animal was handled, 15 min/day, on 2 consecutive days prior to the experiment. All experimental procedures were performed according to the NIH Guide for the Care and Use of Laboratory Animals and all protocols were approved by the Institutional Animal Care and Use Committee of Shin Kong Wu Ho-Su Memorial Hospital (IACUC Approval No: 051220001), Taipei, Taiwan.

### 3.2. Chronic Cocaine Exposure

Initially, and with similar body weight, all thirty-two rats were randomly divided into either a vehicle-treated group (phosphate-buffered saline, PBS) or a cocaine-treated group (Cocaine). The PBS group was injected subcutaneously with 0.5 mL phosphate-buffered saline per day for 3 months and the Cocaine group was injected subcutaneously with cocaine at 10 mg/kg per day for 3 months. The experimental design were performed at the same time with previous study [32] and share vehicle-treated group.

### 3.3. Cardiac Characteristics

At the end of the phosphate-buffered saline or cocaine treatment period, all 32 rats were weighed prior to decapitation. The hearts of all animals were excised and cleaned with distilled H_2_O. The hearts of 8 rats from the PBS group and 8 rats from the Cocaine group were soaked in formalin and examined by Hematoxylin-eosin, DAPI staining and TUNEL assay. The hearts of the remaining 8 PBS group and 8 Cocaine group rats were immediately weighted, then frozen, and further examined by Western blotting. The body weight (BW), the whole heart weight (WHW), and the ratio of the whole heart weight to body weight (WHW/BW) were calculated.

### 3.4. Hematoxylin-Eosin Staining

The hearts were perfused and soaked in formalin and covered with wax. Slides were prepared by deparaffinization and dehydration by passing through a series of graded alcohols (75%, 95% and 100%), 15 min at each concentration. The slides were then dyed with hematoxylin and eosin. After being gently rinsed with water, each slide was soaked in 85% alcohol, 100% alcohol I and II for 15 min each. At the end, they were then soaked in Xylene I and Xylene II. Photomicrographs were obtained using Zeiss Axiophot microscopes.

### 3.5. Terminal Deoxynucleotidyl Transferase Biotin-dUTP Nick End Labeling (TUNEL) Assay and 4′,6-Diamidino-2-phenylindole (DAPI) Staining

After excising and cleaning, the hearts were soaked in formalin, dehydrated through graded alcohols, and embedded in paraffin wax. Sections 0.2 μm thick were prepared from paraffin-embedded tissue blocks. The tissue sections were deparaffinized by immersing in xylene, rehydrated, and incubated in phosphate-buffered saline with 2% H_2_O_2_ to inactivate endogenous peroxidases. The sections were then incubated with proteinase K (20 μg/mL), washed in phosphate-buffered saline, and incubated with terminal deoxynucleotidyl transferase and fluorescein isothiocyanate-dUTP for 60 min at 37 °C, using an apoptosis detection kit (Roche Applied Science, Indianapolis, IN, USA). After washing twice in phosphate-buffered saline, the sections were stained with 4′,6-diamidine-2-phenylindole dihydrochloride (DAPI, Sigma, St. Louis, MO, USA) for 5 min. TUNEL-positive nuclei (fragmented DNA) fluoresce bright green at 450–500 nm, whereas DAPI-positive nuclei (intact DNA) fluoresce blue at 360 nm. The mean number of TUNEL-positive and DAPI-labeled cells was counted in least 5–6 separate fields × 2 slides × 3 left ventricle regions (upper, middle, lower) excised from 6 rat hearts in each group. All counts were performed by at least two independent individuals in a blinded manner.

### 3.6. Tissue Extraction

Cardiac tissue extracts were obtained by homogenizing the left ventricle samples in a lysis buffer (20 mM Tris, 2 mM EDTA, 50 mM 2-mercaptoethanol, 10% glycerol, pH 7.4, proteinase inhibitor cocktail (Roche), phosphatase inhibitor cocktail (Sigma)) at a ratio of 100 mg tissue/1 mL buffer for 1 min. The homogenates were placed on ice for 10 min and then centrifuged twice at 12,000× *g* for 40 min. The supernatant was collected and stored at −70 °C for further investigation.

### 3.7. Separation of Cytosolic and Mitochondrial Fractions

To detect cytosolic cytochrome *c*, tissues were suspended in a buffer (50 mM Tris (pH 7.5), 0.5 M NaCl, 1.0 mM EDTA (pH 7.5), 10% glycerol and proteinase inhibitor cocktail tablet (Roche) for 3 min. on ice), homogenized by 40 strokes in a Dounce homogenizer, and centrifuged at 12,000× *g* for 15 min. The supernatant was the cytosol fraction, and the pellet was resuspended in lysis buffer as the membrane fraction.

### 3.8. Electrophoresis and Western Blot

The tissue extract samples were prepared as described by homogenizing with buffer. Sodium dodecyl sulfate-polyacrylamide gel electrophoresis was run in 10% polyacrylamide gels. The samples were electrophoresed at 140 V for 3.5 h and equilibrated for 15 min in 25 mM Tris-HCl, pH 8.3, containing 192 mM glycine and 20% (*v*/*v*) methanol. Electrophoresed proteins were transferred to polyvinylidene difluoride (PVDF) membrane (Millipore, Bedford, MA, USA, 0.45 μm pore size) with a Bio-rad Scientific Instruments Transphor Unit at 100 V for 2 h. PVDF membranes were incubated at room temperature for 1 h in blocking buffer containing 100 mM Tris-HCl, pH 7.5, 0.9% (*w*/*v*) NaCl. Primary antibodies including TNF-alpha, Fas ligand, Fas receptor, FADD, Bid, t-Bid, Bcl-xL, Bak, Bcl-2, Bax, cytochrome *c*, caspase-8, -9, -3 and α-tubulin (Santa Cruz Biotechnology, Santa Cruz, CA, USA) were diluted to 1:500 in antibody binding buffer containing 100 mM Tris-HCL, pH 7.5, 0.9% (*w*/*v*) NaCl, 0.1% (*v*/*v*) Tween-20 overnight, and incubated overnight at 4 °C. The immunoblots were washed three times in TBS buffer (Tris-Base, NaCl, Tween-20, pH 7.4) for 10 min and then immersed in the second antibody solution containing goat anti-mouse IgG-HRP, goat anti-rabbit IgG-HRP, or donkey anti goat IgG-HRP (Santa Cruz) for 1 hour and diluted 500-fold in TBS buffer. The immunoblots were then washed three times in blotting buffer for 10 min each time. The immunoblotted proteins were visualized by using an enhanced chemiluminescence ECL western blotting Luminal Reagent (Santa Cruz) and quantified using a Fujifilm LAS-3000 chemiluminescence detection system (Tokyo, Japan). Densitometric analysis of immunoblots was performed by an AlphaImager 2200 digital imaging system (Digital Imaging System, San Leandro, CA, USA).

### 3.9. Statistical Analysis

The data from the PBS and Cocaine groups were compared using Student’s *t*-test for two independent samples. In all cases, a difference at *p* < 0.05 was considered statistically significant. Data are presented as mean ± standard deviation (SD).

## 4. Conclusions

This study raises further questions, as to whether anti-apoptotic therapy might be beneficial to attenuate cardiac apoptosis, such as Minocycline [13]. Of course, further therapeutic or clinical studies are required to clarify the effects of treatments or possible mechanisms in chronic cocaine-related heart abnormalities. After integrating our current findings into previously proposed apoptotic theories, our hypothesis proposes that cardiac Fas-dependent and mitochondria-dependent apoptotic pathways might be more activated after three months of cocaine exposure, which might provide a possible mechanism for the development of cardiac abnormality in humans with chronic cocaine abuse.

## Figures and Tables

**Table 1.** Cardiac characteristics of PBS and Cocaine groups.

Cardiac characteristics	PBS	Cocaine
Number of animals	8	8
Body weight (BW), gm	340.70 ± 33.40	319.50 ± 11.80
Whole heart weight (wet WHW), gm	1.51 ± 0.18	1.31 ± 0.18
WHW/BW (×10^4^)	44.76 ± 7.14	41.21 ± 5.80

Values are means ± SD. PBS, Wistar rats with PBS exposure; Cocaine, Wistar rats with cocaine exposure; No significant difference between PBS and Cocaine groups.

## Abbreviations

H&E: Hematoxylin and eosin
DAPI: 4′,6-diamidino-2-phenylindole
TUNEL: Terminal deoxynucleotidyltransferase UTP Nick End Labeling
PBS: phosphate buffered saline, vehicle-treated group
Cocaine: cocaine-treated group
TNF-α: Tumor necrosis factor-alpha
Fas: Fas death receptor
Fas L: Fas ligand
FADD: Fas-associated death domain
Bid: Bcl-2 homology domain 3 (BH3) interacting domain death agonist
t-Bid: truncated Bid
Bcl-xL: B-cell lymphoma-extra large
Bak: Bcl-2 homologous antagonist/killer
Bcl-2: B-cell lymphoma 2
Bax: Bcl-2–associated X protein
Cyt *c*: cytochrome *c*
Bad: Bcl-2–associated death promoter
Cas: caspase
